# A nomogram for predicting prognostic risk factors in individuals with poor grade aneurysmal subarachnoid hemorrhage: a retrospective study

**DOI:** 10.1007/s10143-025-03188-8

**Published:** 2025-01-07

**Authors:** Li Song, Marvin Darkwah Oppong, Philipp Dammann, Karsten H. Wrede, Yahya Ahmadipour, Meltem Gümüs, Thiemo Florin Dinger, Laurèl Rauschenbach, Yan Li, Benedikt Frank, Ulrich Sure, Ramazan Jabbarli

**Affiliations:** 1 Department of Neurosurgery and Spine Surgery, University Hospital Essen, University of Duisburg-Essen, Essen, Germany; 2 Center for Translational Neuro- & Behavioral Sciences (C-TNBS), University of Duisburg Essen, Essen, Germany; 3 Department of Diagnostic and Interventional Radiology and Neuroradiology, University Hospital Essen, University of Duisburg-Essen, Essen, Germany; 4 Department of Neurology, University Hospital Essen, University of Duisburg-Essen, Essen, Germany

**Keywords:** Aneurysmal subarachnoid hemorrhage, Outcome, Risk factors, Prognosis, Nomogram

## Abstract

Poor-grade aneurysmal subarachnoid hemorrhage (PGASAH) is associated with high mortality and morbidity despite advanced treatments. Accurate prediction of prognosis remains a clinical challenge. This study aimed to identify independent risk factors and develop a predictive nomogram for unfavorable outcomes in PGASAH patients. This retrospective study included 348 PGASAH patients treated between 2003 and 2016. Univariate and multivariate logistic regression analyses were performed to evaluate the association of premorbid conditions, admission aSAH clinical parameters, admission laboratory parameters, and clinical complications with unfavorable outcomes, defined as mRS > 2 at 6 months. Independent predictors were identified, and a nomogram was constructed to assess predictive accuracy and clinical utility. The median age of PGASAH patients was 55.1 years, 64.9% were females. Independent predictors of unfavorable outcomes (mRS > 2) at 6 months included age > 55 years (aOR = 2.44), cardiac valve disease (aOR = 6.50), dilated pupils at admission (aOR = 2.64), early infarction (aOR = 5.56), and delayed cerebral ischemia (aOR = 5.09). The nomogram demonstrated good predictive performance with an AUC of 0.807. Decision curve analysis affirmed its clinical applicability. The intuitive nomogram helps physicians predict PGASAH prognosis at discharge, improving long-term management, optimizing resource allocation, and enhancing patient and family awareness and psychological preparedness. External validation is required for broader clinical use.

**Supplementary Information:**

The online version contains supplementary material available at 10.1007/s10143-025-03188-8.

## Introduction

In neurosurgical emergencies, poor-grade aneurysmal subarachnoid hemorrhage (PG[ASAH]) defined as World Federation of Neurosurgical Societies (WFNS) Grade 4–5 represents a critical concern, characterized by high mortality and high disability rate [1, 2]. Despite significant advancements in the diagnosis and treatment of PGASAH in recent years, with surgical interventions playing a pivotal role in enhancing survival rates and quality of life [3], poor-grade aSAH (PGASAH) often results in adverse prognoses, imposing significant burdens on patients and their families [4]. However, the long-term prognosis of surgical patients is influenced by multiple factors, the interplay of which has not been fully elucidated. Accurately predicting and improving long-term quality of life and functional recovery for surgical patients with PGASAH continues to be an unresolved challenge. Given this backdrop, comprehensive prognostic studies are essential to enhance patient outcomes and guide clinicians and families in making informed decisions for these high-risk patients.

Previous research on PGASAH prognosis has centered on short-term outcomes like surgical complications and early survival rates [5, 6], with insufficient exploration of factors affecting long-term functional recovery. Most of the studies have limited sample sizes, restricting generalizability and increasing risk of bias [7]. In addition, the inclusion of PGASAH cases without aneurysm treatment has limited the practical value of these studies for clinicians [8]. Moreover, the current PGASAH prognostic studies investigate a limited number of potential contributing factors, failing to comprehensively capture the risk factors influencing prognosis [9–11].

Nomograms present tools for accurately predicting disease prognosis and are applicable across various pathologies. Crucially, they simplify the complexity of traditional predictive model formulae into a single numerical estimate to predict the likelihood of an event, offering valuable support in clinical decision-making for individual patient situations [12]. Currently, nomograms have widespread clinical applications. To the best of our knowledge, the only existing prognostic study that included a nomogram for patients with PGASAH is the one conducted by Zhou and colleagues in 2023 [13]. However, this study specifically focused on a subset of patients who underwent microsurgical clipping. Consequently, a nomogram representative for the whole PGSAH subpopulation is still lacking.

This study aimed to evaluate the influence of a wide range of potential risk factors that ultimately might impact the outcome of PGASAH undergoing aneurysm treatment.

## Materials and methods

This retrospective analysis included aSAH patients classified as WFNS grades 4–5, defined as PGASH. These patients were admitted between January 2003 and June 2016, with WFNS assessments conducted at the time of admission. The study received approval from the Institutional Review Board (Ethik-Kommission, Medizinische Fakultät der Universität Duisburg-Essen; Approval No. 15–6331-BO) and was registered with the German Clinical Trials Register (DRKS; ID DRKS00008749). WFNS assessments were conducted upon patient admission. All procedures were performed in accordance with relevant guidelines and regulations, and informed consent was obtained from all participants or their legal guardians.

### Study population

We identified patients with PGASAH through screening of our electronic medical database. Diagnosis of aSAH was made at admission based on computed tomography (CT) scans and/or lumbar puncture, demonstrating xanthochromic cerebrospinal fluid (CSF) indicative of subarachnoid blood, and proof of an intracranial aneurysm by angiogram. For the purposes of this study, clinical grade was recorded based on the patients' status at admission, prior to any interventions such as external ventricular drainage (EVD), medication, surgical treatment, or other therapeutic measures. Inclusion required patients aged ≥ 18 years who underwent aneurysm treatment in our neurosurgical department. Exclusion criteria included: 1) age < 18 years, 2) absence of aneurysm treatment, 3) lack of 6-month postoperative follow-up records.

### Clinical therapeutic protocol

All patients admitted with suspected aSAH undergo radiographic imaging, including digital subtraction angiography (DSA) or CT angiography, to ascertain the source of bleeding. Treatment decisions, determined through collaborative assessments by the on-call neuroradiologist and neurosurgeon, involved microsurgical clipping or endovascular coiling. All patients received oral nimodipine for 21 days post-onset. Our treatment and management protocols strictly followed the international guidelines set forth by the American Heart Association/American Stroke Association (AHA/ASA) 2012 for the management of aSAH, ensuring standardized, evidence-based care for PGASAH patients [14].

For patients presenting with clinical or radiological evidence of elevated ICP (e.g., acute hydrocephalus, brain herniation, or severe neurological deterioration), ICP was monitored using EVD to ensure accurate measurements. Patients with clinical or radiological evidence of acute hydrocephalus underwent EVD insertion as part of standard management. For patients without EVD placement, ICP was assessed using radiological criteria, including compressed basal cisterns, midline shift, or significant ventricular dilation consistent with acute hydrocephalus. Patients with stable radiological dynamics were classified as having ICP ≤ 20 mmHg. Conservative treatment for ICP was initiated if ICP remained persistently elevated (> 20 mmHg), while decompressive craniectomy was considered for refractory cases. Transcranial Doppler ultrasound (TCD) was conducted daily for at least 14 days post-onset to identify cerebral vasospasm. Patients exhibiting clinical symptoms of vasospasm (TCD values > 120 m/s, new neurological deficit, or Glasgow Coma Scale (GCS) decline ≥ 2 points) were scheduled for DSA for verification and invasive endovascular treatment. Routine laboratory tests were conducted at admission and three times a week during stay on intensive care unit, with additional blood tests as necessary.

Additional routine CT scans were performed within the first 24 h post-treatment, after any further surgical interventions, or in case of clinical deterioration. Close monitoring of vital parameters in the intensive care unit or intermediate care unit was mandatory for at least 14 days post-aSAH.

### Patient demographics and clinical parameters

Data on demographics, admission comorbidities, previous medication history, admission aSAH-related parameters, laboratory parameters, and complications during hospitalization were collected from electronic patient records, standardized admission protocols, and intensive care charts.

For statistical analysis, patients' age was dichotomized (≤ 55 years vs. > 55 years), corresponding to the cohort's median age. The size of the aneurysm sac was measured using DSA, and was categorized at a cutoff of 6 mm. Aneurysms exhibiting multiple lobes or irregular shapes were designated as "irregular aneurysm morphology." Initial clinical severity was quantified using the WFNS grading scale [15]. The extent of hemorrhage was assessed via the original Fisher scale [16], segregating severity into low (Fisher grades 1–2) and high (Fisher grades 3–4) categories.

Intracranial aneurysms are divided into anterior circulation aneurysms and posterior circulation aneurysms. Anterior circulation aneurysms include aneurysms that occur in a series of internal carotid artery systems, including the carotid artery, ophthalmic artery, choroidal artery, posterior communicating artery, middle cerebral artery, anterior cerebral artery, and anterior communicating artery. Posterior circulation aneurysms are aneurysms that occur in the vertebrobasilar artery, such as basilar artery aneurysms, posterior cerebral artery aneurysms, vertebral artery or superior cerebellar artery aneurysms, posterior cerebellar or anterior inferior cerebellar artery aneurysms. The incidences of both intracerebral (ICH) and intraventricular hemorrhages (IVH) were meticulously documented as well as laboratory parameters at admission.

Admission aSAH-related parameters included time from ictus to treatment (in days), Fisher grade 3–4, dilated pupil(s) at admission, and admission ICP > 20 mmHg. During statistical analysis, ICP values obtained via EVD were used as precise measurements, while radiological data were utilized for patients without invasive monitoring. For simplicity, ICP values were categorized dichotomously as either ≤ 20 mmHg or > 20 mmHg for statistical evaluation. Treatment modalities, including coiling or clipping, were documented, along with aneurysm location (anterior vs. posterior circulation), presence of IVH, ICH, and ICH evacuation. Additional parameters such as aneurysm size > 6 mm, multiple aneurysms, irregular aneurysm morphology, and admission maximum temperature (in Celsius) were also recorded.

Information regarding comorbidities such as obesity, drug abuse, alcohol abuse, hypercholesterolemia, diabetes, oncologic, hypothyroidism, hyperthyroidism, hyperuricemia/Gout, and cardiac valve disease, as well as previous medication history including beta blocker, calcium channel blocker, ACE inhibitor, AT1 antagonist, statin, acetylsalicylic acid (ASA), and warfarin, were extracted from patients' original medical records. Cardiac valve disease was defined as a history of clinically diagnosed valvular disease documented in the medical records or echocardiography findings, including valve stenosis, insufficiency, or other significant structural abnormalities. There were no patients with a history of valve replacement surgery in the final analysis.

We also recorded the complications during the acute hospitalization, including aneurysm rebleeding, CNS infection, decompressive craniectomy (DC), increased ICP, TCD > 120 m/s, angiographic vasospasm, systemic infection, sepsis, pneumonia, pleural effusion, pneumothorax, tracheotomy, bacteremia, acute coronary syndrome, new onset of arrhythmia, thrombotic complexes, gastrointestinal complication, liver dysfunction, epilepsy, seizure at onset (SaO), DCI infarction and early infarction. New low-density areas seen in follow-up CT scans, not attributable to surgical intervention or ICH, were classified as cerebral infarcts. Infarcts documented within 72 h post-hemorrhage were categorized as early infarctions [17], while those occurring subsequently were defined as delayed cerebral ischemia (DCI) [18].

### Outcome

All outcomes were assessed by professional physicians using the modified Rankin Scale (mRS) [19] during the 6-month follow-up. The primary endpoint of this study was defined as an unfavorable outcome, characterized by an mRS score of 3 or higher at the 6-month follow-up. Functional disability was defined by mRS score ≥ 2. In-hospital mortality was documented.

### Statistical analysis

Statistical analyses were conducted using SPSS Version 26 for Mac (IBM Corp.). The significance threshold was established at p < 0.05. If the count of missing values was less than 18, the complete-case analysis method was utilized, focusing solely on individuals with complete data across all variables. For counts equal to or exceeding 18, a univariate imputation approach was adopted. Categorical variables were imputed with mode values, while quantitative variables were imputed with means for analysis. Initially, a univariate analysis of all collected parameters was conducted to discern their impact on prognostic outcomes. Dichotomous variables were evaluated using Chi-square tests or Fisher's exact test for sample sizes less than five. Continuous variables were analyzed using Student's t-test for normally distributed data and the Mann–Whitney U test for non-normal distributions. Cut-off values for continuous variables were identified through receiver operating characteristic (ROC) curve analysis using the Youden index, aiming to maximize sensitivity and specificity. These thresholds were applied as criteria before inclusion in the multivariate analysis. Subsequently, significant variables (p < 0.05) from univariate analyses were incorporated into a multivariate binary logistic regression model to identify independent prognostic predictors. This multivariate analysis was executed in a staged approach: first, analyzing variables from premorbid conditions/medications, admission aSAH dependent/clinical parameters, admission laboratory parameters, and aSAH/clinical complications separately, and then integrating the significant variables from these analyses into the final multivariate model. Missing data were replaced using multiple imputation.

R version 4.3.0 (2023–04–21) was employed to construct prognostic nomograms integrating these independent risk factors. ROC curves were used to assess the discriminative ability of the nomograms. The area under the curve (AUC) represents the area covered by the ROC curve, with an AUC value approaching 1 indicating enhanced predictive efficacy. An AUC > 0.70 suggests acceptable model discrimination. Calibration curves were employed to assess the accuracy of the predictive model in estimating individual prognostic outcomes. Decision curve analysis (DCA) was utilized to evaluate the clinical utility of the prognostic model.

## Results

### Demographic and clinical characteristics of patients

**Fig. 1:**
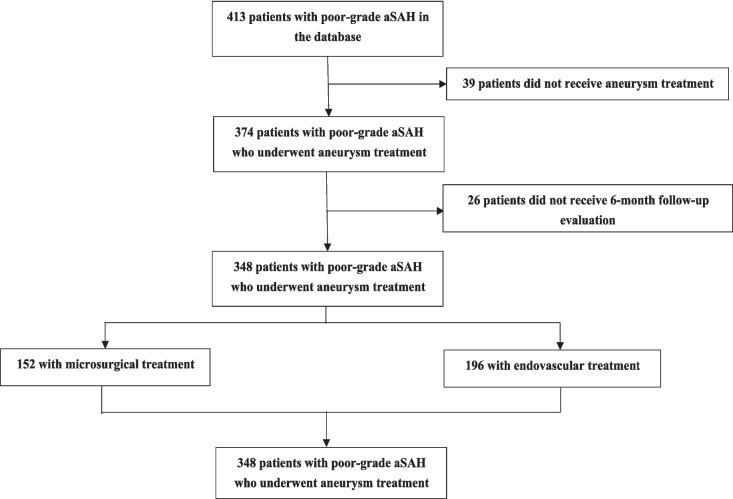
Flow diagram of the study recruitment

During the interval from January 2003 to June 2016, our institution documented the admission of 413 individuals diagnosed with PGASH. Of these, 374 patients (90.6%) underwent aneurysm-related endovascular or microsurgical interventions. A subset of 26 patients was excluded from the analysis due to the loss of follow-up data. Ultimately, 348 (84.3%) patients with PGASH who received initial treatment were included in this study (Fig. 1). Additionally, 230 (66.1%) PGASAH patients were classified as WFNS grade 4, and 118 (33.9%) PGASAH patients were classified as WFNS grade 5.

The majority of these patients (56.3%) underwent endovascular coiling, whereas 43.7% received microsurgical clipping. The demographic profile was predominantly Caucasian (95.7%) with an average age of 55.1 years (ranging from 24 to 87 years). Females comprised 64.9% of the study population. Notably, a significant proportion had a medical history of hypertension (70.7%, Table 1).

**Table 1:** Baseline and premorbid diseases characteristics of the 348 included patients

	PGASAH patients undergoing surgery	
Characteristic	n/mean	%/SD
No. of pts	348	/
Age (years, median with IQR)	55.1	13.0
Sex(Female)	226	64.9%
Ethnicity (Caucasian)	333	95.7%
Arterial hypertension	246	70.7%
Alcohol abuse	24	6.9%
Drug abuse	9	2.6%
Obesity	23	6.6%
Hypercholesterolemia	20	5.7%
Hypothyroidism	37	10.6%
Hyperthyroidism	4	1.1%
Hyperuricemia	5	1.4%
Cardiac valve disease	39	11.2%
Diabetes	21	6.0%
Oncologic diseases	20	5.7%
Peripheral artery occlusive disease	3	0.9%
NSAID	14	4.0%
Chronic inflammation	17	4.9%
Beta-blocker	55	15.8%
Calcium channel blocker	31	8.9%
ACE inhibitor	70	20.1%
AT1 antagonist	21	6.0%
Statin	14	4.0%
ASA	21	6.0%
Warfarin	5	1.4%
Fisher grade 3–4	340	97.7%
Dilated pupil(s) at admission	53	15.2%
In hospital mortality	95	27.3%
Unfavorable outcome at 6 months	219	62.9%

Upon prognostic evaluation of the cohort, in-hospital mortality occurred in 27.3% (95/348) of the patients. At the 6-month follow-up examination, a considerable number of individuals (62.9%) demonstrated unfavorable outcomes (Table 1). The treatment modality did not exhibit a statistically significant impact on in-hospital mortality rates and unfavorable outcomes at the 6-month (Supplementary Table 1).

### Univariate analysis of prognostic factors

We utilized univariate analysis to evaluate the risk factors for unfavorable outcome at 6-month follow-up. In the premorbid conditions group, factors such as age > 55, female sex, hypothyroidism, cardiac valve disease, and statin medication usage demonstrated significant prognostic value. Within the initial aSAH characteristics, significant differences were noted for Fisher Grade 3–4, IVH, ICH, aneurysm size > 6 mm, the presence of dilated pupil(s), and increased ICP, while no statistically significant association between acute hydrocephalus and unfavorable outcomes at 6 months was observed. In the admission laboratory parameters group, initial creatinine levels, glucose levels, and albumin levels showed a significant impact on the outcome. In the group of adverse events during aSAH, preoperative aneurysmal rebleeding, DC, increased ICP, early infarction, and DCI exhibited significant differences (Supplementary Table 2).

### Multivariate analysis of prognostic factors

Subsequently, variables that showed significant differences in the univariate analysis were then included in the primary multivariate logistic regression analysis within their groups. Laboratory data identified as significant in univariate analysis were dichotomized before being incorporated into the multivariate analysis. ROC analysis identified cut-offs for creatinine (1.002 mg/24 h, AUC = 60.3, p = 0.005), glucose (127.5 mg/dL, AUC = 64.5, p = 0.001), and albumin (13.5 g/L, AUC = 60.6, p = 0.004) (Supplementary Fig. 1). For clinical applicability, these values were rounded to creatinine > 1 mg/24 h, glucose > 130 mg/dL, and albumin > 14 g/L. The first round of multivariate analysis identified the following prognostic indicators: age > 55 years, female sex, hypothyroidism, cardiac valve disease, dilated pupil(s) at admission, IVH, albumin > 14 g/L, DCI, and early infarction (Table 2). Integration of these factors into a final multivariate logistic model elucidated several critical independent risk factors relevant to prognosis. Finally, PGASAH individuals aged over 55 years faced a two-fold increase in the risk of unfavorable outcomes (adjusted odds ratio [aOR] = 2.44; 95% confidence interval [CI]: 1.37–4.34). The presence of cardiac valve disease (aOR = 6.50; 95% CI: 1.60–19.04) and dilated pupil(s) at admission (aOR = 2.64; 95% CI: 1.13–6.16) also negatively impacted the outcome. Accordingly, PGASAH individuals without these three admission risk factors (higher age, cardiac valve diseases, and dilated pupils(s)), had a 50% chance of achieving a favorable outcome 6-month post-aSAH (mRS = 2 and below, Supplementary Table 3 and Supplementary Fig. 2). Finally, the occurrence of early infarction (aOR = 5.56; 95% CI: 3.10–9.97) and DCI (aOR = 5.09; 95% CI: 2.74–9.44) during treatment were confirmed as independent predictors of long-term unfavorable outcome after PGASAH.

**Table 2:** Multivariate analysis of factors contributing to poor prognosis. (**a**. Premorbid conditions/medications, **b**. Admission aSAH dependent/clinical parameters, **c**. Admission laboratory parameters, **d**. aSAH(clinical complications, **e**. Combined final MVA including all parameters with p < 0.05 from the first round)

Parameter	*p*	aOR	95%CI(Lower–Upper)
Age > 55 years	**0.005**	2.00	1.23–3.23
Female sex	**0.047**	0.60	0.36–1.99
Hypothyroidism	**0.043**	0.47	0.22–0.98
Cardiac valve disease	**0.007**	5.52	1.60–19.04
Statin therapy	0.124	5.60	0.63–50.07
a. Premorbid conditions/medications			
Parameter	*p*	aOR	95%CI(Lower–Upper)
Fisher 3–4	0.873	1.19	0.13–10.67
Dilated pupil(s)	**0.007**	2.29	1.34–6.26
Admission ICP > 20 mmHg	0.108	1.51	0.91–2.49
IVH	** < 0.001**	2.64	1.59–4.37
ICH	0.118	1.49	0.90–2.46
Aneurysm size > 6 mm	0.129	1.44	0.90–2.32
b. Admission aSAH clinical and radiographic parameters			
Parameter	*p*	aOR	95%CI(Lower–Upper)
Creatinine > 1(mg/24 h)	0.141	1.57	0.86–2.87
Glucose > 130(mg/dL)	0.112	1.95	0.84–4.54
ALB > 14 (g/L)	**0.047**	1.80	1.01–3.20
c. Admission laboratory parameters			
Parameter	*p*	aOR	95%CI(Lower–Upper)
Aneurysm rebleeding	0.121	2.54	0.78–8.27
Decompressive craniectomy	0.089	1.60	0.93–2.74
Increased ICP	0.228	1.40	0.81–2.40
DCI Infarction	** < 0.001**	4.19	2.32–7.59
Early Infarction	** < 0.001**	4.48	2.63–7.64
d. Adverse events during aSAH			
Parameter	*p*	aOR	95%CI(Lower–Upper)
Age > 55 years	**0.002**	2.44	1.37–4.34
Female sex	0.171	0.60	0.37–1.19
Hypothyroidism	0.063	0.43	0.22–0.98
Cardiac valve disease	**0.004**	6.50	1.60–19.04
Dilated pupil(s)	**0.025**	2.64	1.13–6.16
IVH	0.079	1.65	0.94–2.89
ALB > 14(g/L)	0.600	1.17	0.65–2.13
DCI infarction	** < 0.001**	5.09	2.74–9.44
Early infarction	** < 0.001**	5.56	3.10–9.97
e. Combined final MVA including all parameters with p<0.05 from the first round			

### Prognostic modeling and risk stratification

**Fig. 2:**
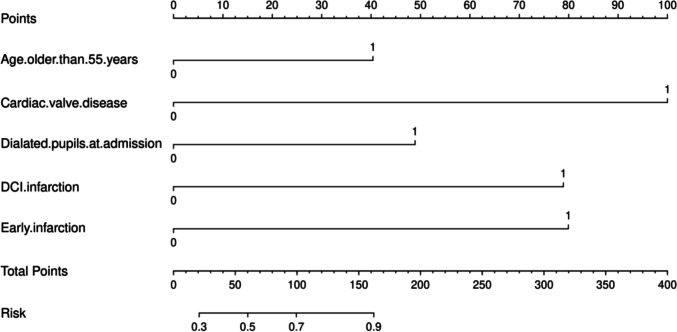
The prediction nomograms of risk factors for poor prognosis in poor-grade aSAH patients

**Fig. 3:**
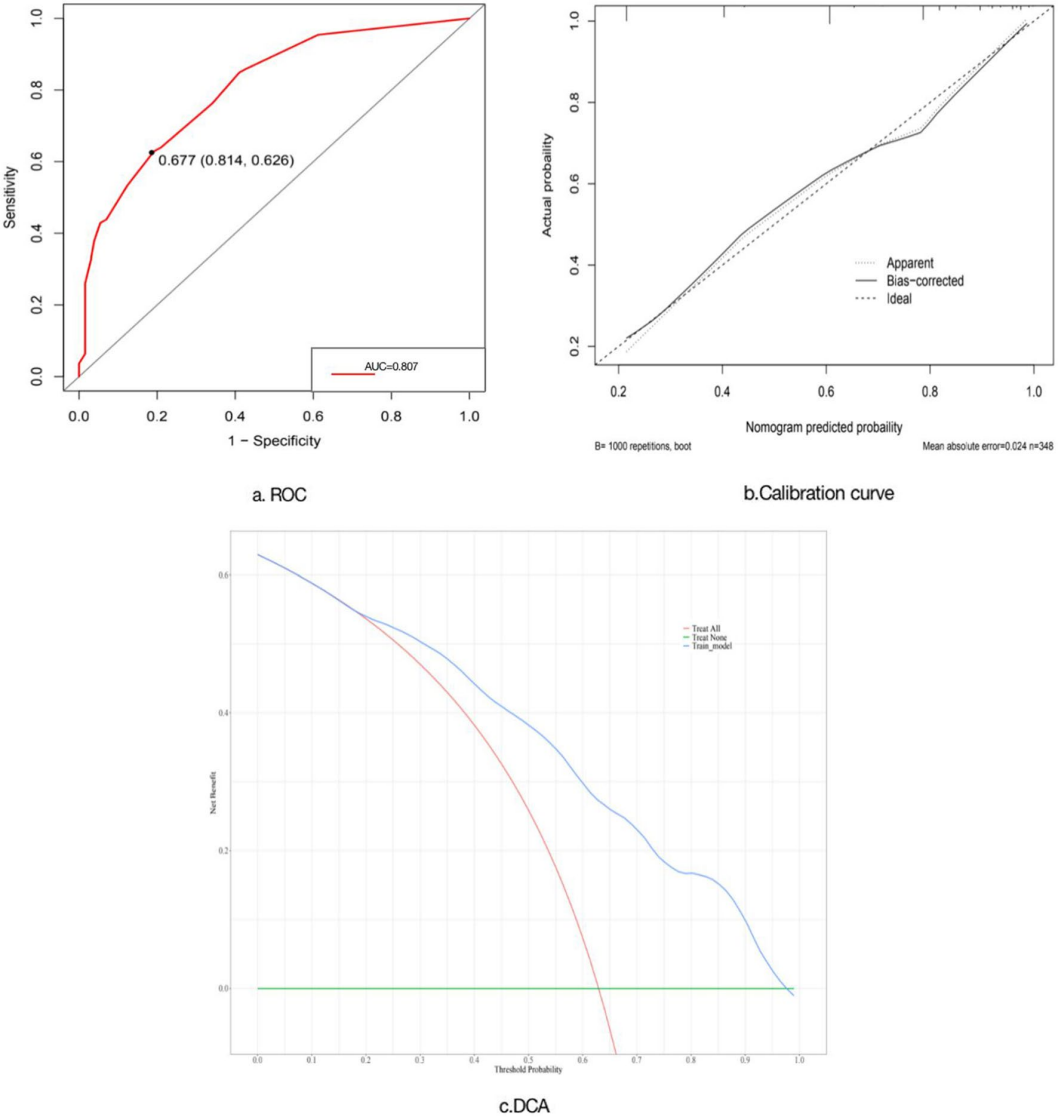
Performance evaluation of predictive models **a**. Differentiation: Describing the ability of a model to correctly distinguish between corresponding clinical outcomes and non-corresponding clinical outcomes, represented by consistency index (CI), essentially equal to the area AUC under the Receiver operating characteristic (ROC) curve. **b**. Calibration: describes the accuracy of a model in predicting the probability of individual clinical outcomes. Use a calibration curve to evaluate the calibration degree. A straight line with a slope of 1 is the ideal curve. The closer the actual curve is to the ideal curve, the smaller the deviation between the predicted results and the actual results of the model, and the better the model performance. **c**. Decision curve analysis (DCA): evaluating the Clinical Practicality of Models

Based on the results of multivariate analysis, we constructed a nomogram, as illustrated in Fig. 2. In this model, the incorporated risk factors were assigned differential weights corresponding to their respective degrees of impact. These factors were then quantitatively scored based on individual patient data. The cumulative scoring provided an aggregate score, facilitating prognostic predictions that are visually represented and easily interpretable in the constructed nomogram. The model's predictive precision and discriminative capacity were gauged using the Concordance Index (C-index) and calibration curves. Based on ROC curve analysis (Fig. 3), the nomogram's C-index was 0.807 (95% CI: 0.761–0.853), demonstrating robust predictive accuracy. Calibration curves indicated a strong concordance between predicted and observed outcomes, affirming the nomogram's reliability. Furthermore, the clinical utility of the nomogram was assessed using DCA, suggesting its significant contribution to clinical predictive accuracy.

## Discussion

Among aSAH patients, PGASAH demonstrates an even higher mortality and disability rate. Despite adhering to international guidelines (AHA/ASA 2012) and providing active treatment and management for PGASAH patients, 27.3% of PGASAH individuals in our study showed in-hospital mortality, 71.3% functional disability at 6-month post-surgery, and 62.9% an overall unfavorable outcome. These findings underscore the persistent neurological deficits and significant societal and familial impacts on these patients [20–22]. Thus, our study aimed at a comprehensive and systematic exploration of prognostic factors affecting PGASAH patients, particularly quantifying these factors in a multivariate context.

Notably, despite 56.3% of patients undergoing endovascular coil embolization and 43.7% undergoing microsurgical clipping, the choice between treatment modalities (clipping vs. coiling) did not statistically impact in-hospital mortality, functional disabilities, or unfavorable outcomes 6-month after treatment. This finding aligns with the results of the study by Shen and colleagues [23], but challenges the conventional notion that the selection of surgical techniques might be the determining factor in the prognosis of aSAH or PGASAH cases [24, 25]. This discovery significantly contributes to the ongoing debate in neurosurgical practice, indicating the necessity for an individualized approach based on patient-specific factors rather than a preference for a particular technique.

Age, brain herniation, WFNS V grade, and higher modified Fisher scores are key contributors to unfavorable outcomes in aSAH patients [11, 26–28]. Our study reaffirms the age-related risk, highlighting that patients aged over 55 years are twice as likely to experience adverse outcomes compared to their younger counterparts. This resonates with prior research indicating that elderly individuals [29, 30], even within favorable aSAH grades [31, 32], experience poorer prognoses. This is attributed to their compromised vascular health and diminished capacity to recover from initial hemorrhage events [33, 34]. The higher frequency of cardiac valve disease in the mRS 3–6 group (17%) likely reflects the older age distribution and increased prevalence of comorbidities in poor-grade aSAH patients. Structural valve disease is well-documented to increase with age [35, 36], and this underscores the interplay between advanced age, comorbidities, and poor outcomes in PGASAH patients.

Our study also focused on patient comorbidities, identifying cardiac valve disease in 11% of our cohort, with a particularly strong association with unfavorable outcomes—an observation not previously reported in PGASAH research. This finding suggests that cardiac valve disease may play an underappreciated role in the pathophysiology and prognosis of PGASAH. Previous studies have primarily emphasized cardiac complications arising after SAH [37, 38], such as catecholamine-induced myocardial dysfunction or neurogenic stunned myocardium, triggered by excessive sympathetic activation following SAH [39–42]. Up to 50% of SAH patients experience such cardiac dysfunction, which has been independently linked to an increased risk of DCI, cerebral infarction, and poor outcomes due to impaired systemic hemodynamics and cerebral perfusion [43–45]. In our study, compromised cardiac output in patients with cardiac valve disease may have exacerbated hemodynamic instability, increasing the likelihood of cerebral hypoperfusion and secondary ischemic injuries such as DCI, which was also identified as an independent prognostic factor. Interestingly, to ensure cohort homogeneity, we excluded patients with prosthetic valve replacements, thereby minimizing the confounding effects of warfarin use on intracranial hemorrhage outcomes. This highlights the distinct impact of native valve disorders on PGASAH prognosis. These findings underscore the importance of close monitoring and personalized management for PGASAH patients with cardiac valve disease.

In PGASAH patients, relying solely on the WFNS grading system for clinical assessment and prognosis has limited effectiveness [46]. Our study expanded on PGASAH patients by considering brainstem dysfunction signs, such as abnormal pupils (anisocoria or bilateral dilation). We found that PGASAH patients with abnormal pupils had notably worse outcomes. This finding aligns with studies by Kobata, Raabe, and others, indicating that a worse WFNS or GCS grade combined with abnormal pupils may predict extremely high mortality rates and unfavorable outcomes [46, 47]. Our study also affirms the pivotal role of dilated pupil(s) at admission in assessing the prognosis of PGASAH. Acute hydrocephalus is a frequent clinical condition in patients with PGASAH, often requiring EVD to manage elevated intracranial pressure. Although we hypothesized that acute hydrocephalus could independently affect prognosis, our analysis did not find a statistically significant association between acute hydrocephalus and unfavorable outcomes. This suggests that timely and effective management of elevated ICP might mitigate its impact. However, further research is needed to explore the effects of acute hydrocephalus stratified by EVD response and to refine prognostic models for PGASAH patients.

Additionally, our study emphasized complications during treatment, revealing that both early cerebral infarction and DCI significantly increase the risk of unfavorable outcomes. Early cerebral infarction, occurring within 72 h after SAH onset, directly damages brain tissue and constitutes a devastating neurological event [17, 48, 49]. DCI, a common SAH complication occurring after 72 h, caused by vasospasm or a new cerebral infarction related to vasospasm [17]. Our findings align with previous research, emphasizing DCI as an independent predictor of adverse outcomes at 6-month post-vascular intervention in elderly aSAH patients [50, 51]. While research on the early infarction-prognosis relationship is limited, a few studies have suggested its association with unfavorable outcomes at 3-month [52–54]. Our study contributes to this evidence, emphasizing the need for meticulous management and attention to early and delayed cerebral infarctions during clinical care. Reducing the occurrence of early cerebral infarction and DCI during hospitalization could lead to improved prognosis.

Nomograms have become popular in prognostic medicine due to their user-friendly interface and clear presentation of prognosis information [55]. Our study is the first to employ a nomogram for assessing the risk of unfavorable outcomes in patients with PGASAH undergoing aneurysm clipping or coiling. By utilizing multivariable logistic regression, we identified age > 55 years, cardiac valve disease, dilated pupil(s) at admission, early infarction, and DCI as independent risk factors. These factors were used to create a predictive tool for determining adverse outcomes 6-month post-surgery. The results demonstrated that the nomogram based on these 5 parameters has robust predictive capabilities for unfavorable outcomes in PGASAH at 6-month after aneurysm treatment. Unlike traditional scoring systems like WFNS or GCS, which primarily focus on single-dimension factors, our model integrates multiple independent predictors to enhance the accuracy of risk stratification. This approach not only optimizes resource allocation and personalized intervention strategies but also empowers patients and their families to better understand the prognosis and prepare psychologically, facilitating informed decision-making.

## Limitations

Certainly, this study has limitations. Being a retrospective, single-center study, it may exhibit selection bias in participant recruitment. It primarily focused on a homogeneous Caucasian population, limiting the generalizability. Moreover, the selection of variables included in the analysis was constrained by the inherent challenges of retrospectively collecting these data. Additionally, while we included ICP measurements for patients with invasive monitoring via EVD, ICP status for those without EVD placement was inferred from radiological data. This approach, while pragmatic, introduces potential variability in ICP classification and may underrepresent dynamic intracranial changes. And we recorded WFNS grading based on patients' initial admission condition, prior to any interventions such as EVD, medication, or surgery. While this approach captures the immediate impact of severe aSAH presentation, it does not account for potential rapid improvements following EVD or ICP management. The lack of external validation reduces the model's credibility. The use of retrospective data from January 2003 to June 2016 introduces certain limitations. While advancements in neurosurgical techniques have remained relatively stable over the past decade, they may still impact the applicability of the findings. Future research should use prospective, multicenter designs with diverse populations and standardized data collection, including comprehensive documentation of comorbidities and complications. External validation of the model is essential to confirm its robustness, and refining subgroup stratification, such as for patients with acute hydrocephalus requiring EVD, could enhance predictive accuracy and provide targeted guidance for personalized management.

Despite these limitations, this study serves as an exploratory effort to develop a comprehensive prognostic framework for PGASAH patients. Despite using older data, it is the first to compile such a large database of PGASAH patients to date. Moreover, our analysis is based on a multivariable prognostic model that incorporates a broad range of potential risk factors, providing a robust and comprehensive assessment. Importantly, this is the first study to employ a nomogram for quantifying and visualizing postoperative prognostic factors in PGASAH, offering clinicians a valuable tool for personalized risk assessment and decision-making. Additionally, the model’s potential to guide post-discharge rehabilitation strategies and address the current gap in long-term management for PGASAH patients highlights its practical value.

## Conclusions

In summary, this study provides an exploratory framework for improving risk stratification in PGASAH patients by analyzing a large cohort of 348 individuals and incorporating established and emerging prognostic factors. Age > 55, cardiac disease, dilated pupil(s) at admission, early infarction, and DCI were identified as independent risk factors for unfavorable outcomes. The proposed prognostic model highlights the importance of individualized patient assessment and offers a valuable tool for predicting and managing PGASAH patients. Further validation in larger, multicenter cohorts is required to confirm its clinical utility and applicability.

## Supplementary Information

Below is the link to the electronic supplementary material.

ESM 1 (DOCX 440 KB)

## Data Availability

The data that support the findings of this study are available on request from the corresponding author. The data are not publicly available due to privacy or ethical restrictions.
