# Supplementary material for: A nomogram for predicting prognostic risk factors in individuals with poor grade aneurysmal subarachnoid hemorrhage: a retrospective study
Source: Neurosurg Rev. 2025 Jan 7;48(1):25. doi: 10.1007/s10143-025-03188-8 (PMC11706874; doi:10.1007/s10143-025-03188-8)

**Supplementary Table 1** Baseline and premorbid diseases characteristics of the 348 included patients compared by treatment method

|  | poor-grade aSAH patients undergoing surgery | | | |
| --- | --- | --- | --- | --- |
| Characteristic | All | coiling | clipping | *p* |
| No. of pts | 348 | 196(56.3%) | 152(43.7%) | / |
| Age (years, median with IQR) | 55.1(24-87) | 55.3(28-87) | 54.8(24-87) | 0.103 |
| Sex(Female) | 226(64.9%) | 126(64.3%) | 100(65.8%) | 0.771 |
| Caucasian race | 333(95.7%) | 190(96.9%) | 143(94.1%) | 0.193 |
| Arterial hypertension | 246(70.7%) | 143(73.0%) | 103(67.8%) | 0.291 |
| Alcohol abuse | 24(6.9%) | 16(8.2%) | 8(5.3%) | 0.283 |
| Drug abuse | 9(2.6%) | 5(2.6%) | 4(2.6%) | 0.970 |
| Obesity | 23(6.6%) | 12(6.1%) | 11(7.2%) | 0.689 |
| Hypercholesterolemia | 20(5.7%) | 8(4.1%) | 12(7.9%) | 0.133 |
| Hypothyroidism | 37(10.6%) | 21(10.7%) | 16(10.5%) | 0.955 |
| Hyperthyroidism | 4(1.1%) | 4(2.0%) | 0 | 0.077 |
| Hyperuricemia | 5(1.4%) | 3(1.5%) | 2(1.3%) | 0.862 |
| Cardiac valve disease | 39(11.2%) | 20(10.2%) | 19(12.5%) | 0.513 |
| Diabetes | 21(6.0%) | 10(5.1%) | 11(7.2%) | 0.414 |
| Oncologic diseases | 20(5.7%) | 14(7.1%) | 6(3.9%) | 0.199 |
| Peripheral artery occlusive disease | 3(0.9%) | 0 | 3(2.0%) | **0.049** |
| Chronic Pain/NSAID | 14(4.0%) | 9(4.6%) | 5(3.3%) | 0.541 |
| Chronic inflammation | 17(4.9%) | 8(4.1%) | 9(5.9%) | 0.437 |
| Beta blocker | 55(15.8%) | 33(16.8%) | 22(14.5%) | 0.546 |
| Calcium channel blocker | 31(8.9%) | 18(9.2%) | 13(8.6%) | 0.836 |
| ACE inhibitor | 70(20.1%) | 39(19.9%) | 31(20.4%) | 0.911 |
| AT1 antagonist | 21(6.0%) | 12(6.1%) | 9(5.9%) | 0.936 |
| Statin | 14(4.0%) | 5(2.6%) | 9(5.9%) | 0.113 |
| ASA | 21(6.0%) | 10(5.1%) | 11(7.2%) | 0.405 |
| Warfarin | 5(1.4%) | 3(1.5%) | 2(1.3%) | 0.869 |
| Fisher grade 3-4 | 340(97.7%) | 189(96.4%) | 151(99.3%) | 0.072 |
| Dilated pupil(s) at admission | 53(15.2%) | 23(11.7%) | 30(19.7%) | **0.039** |
| In hospital mortality | 95(27.3%) | 60(30.6%) | 35(23.0%) | 0.115 |
| Unfavorable outcome at 6 months | 219 (62.9%) | 121(61.7%) | 98(64.5%) | 0.600 |

**Supplementary Table 2** Univariate analysis of premorbid conditions/medications, admission aSAH dependent/clinical parameters, admission laboratory parameters, and aSAH/clinical complicationq’as and 6-month postoperative prognosis in 348 poor-grade aSAH patients

|  | mRS 0-2 | mRS 3-6 |  |  |
| --- | --- | --- | --- | --- |
| **Characteristic** | n=129 | n=219 | *p* Value | missing |
| ***Premorbid conditions/medications*** | | | | |
| Age > 55 years | 45(34.9%) | 116(53.0%) | **0.001** | 0 |
| Female sex | 93(72.1%) | 133(60.7%) | **0.032** | 0 |
| Non-Caucasian | 5(3.9%) | 10(4.6%) | 0.759 | 0 |
| Arterial hypertension | 87(67.4%) | 159(72.6%) | 0.307 | 0 |
| Alcohol abuse | 11(8.6%) | 13(6.1%) | 0.391 | 8 |
| Drug abuse | 3(2.3%) | 6(2.8%) | 0.787 | 8 |
| Obesity | 7(5.5%) | 16(7.5%) | 0.460 | 8 |
| Hypercholesterolemia | 4(3.1%) | 16(7.5%) | 0.093 | 8 |
| Hypothyroidism | 20(15.5%) | 17(7.8%) | **0.024** | 0 |
| Hyperthyroidism | 2(1.6%) | 2(0.9%) | 0.590 | 0 |
| Hyperuricemia/Gout | 0(0.0%) | 5(2.4%) | 0.080 | 8 |
| Cardiac valve disease | 3(2.3%) | 36(17.0%) | **<0.001** | 8 |
| Diabetes | 5(3.9%) | 16(7.3%) | 0.191 | 1 |
| Oncologic diseases | 4(3.1%) | 16(7.5%) | 0.093 | 8 |
| Peripheral artery occlusive disease | 0(0.0%) | 3(1.4%) | 0.176 | 8 |
| NSAID | 6(4.7%) | 8(3.8%) | 0.688 | 9 |
| Beta blocker | 15(11.7%) | 40(18.8%) | 0.086 | 7 |
| Calcium channel blocker | 11(8.6%) | 20(9.4%) | 0.804 | 7 |
| ACE inhibitor | 25(19.5%) | 45(21.1%) | 0.724 | 7 |
| AT1 antagonist | 9(7.0%) | 12(5.6%) | 0.603 | 7 |
| Statin | 1(0.8%) | 13(6.1%) | **0.016** | 7 |
| ASA | 4(3.1%) | 17(7.8%) | 0.079 | 2 |
| Warfarin | 2(1.6%) | 3(1.4%) | 0.888 | 2 |
| ***Admission aSAH clinical and radiographic parameters*** | | | | |
| Time ictus to treatment (days) | 0.876±4.128 | 0.663±1.644 | 0.499 | 0 |
| Fisher 3-4 | 124(94.7%) | 241(99.2%) | **0.007** | 0 |
| Dilated pupil(s) | 9(7.0%) | 44(20.1%) | **0.001** | 0 |
| Admission ICP>20mmHg | 67(51.9%) | 138(63.0%) | **0.043** | 0 |
| Treatment modalities |  |  | 0.600 | 0 |
| Coiling | 75(58.1%) | 121(55.3%) |  |  |
| Clipping | 54(41.9%) | 98(44.7%) |  |  |
| Aneurysm location: |  |  | 0.183 | 0 |
| anterior circulation | 90(69.8%) | 167(76.3%) |  |  |
| posterior circulation | 39(30.2%) | 52(23.7%) |  |  |
| IVH | 72(55.8%) | 162(74.7%) | **<0.001** | 2 |
| ICH | 52(40.3%) | 119(54.3%) | **0.011** | 0 |
| ICH evacuation | 31(25.4%) | 61(30.8%) | 0.300 | 28 |
| Aneurysm size > 6mm | 53.6% | 42.1% | **0.040** | 9 |
| Multiple aneurysms | 51(39.5%) | 78(35.6%) | 0.465 | 0 |
| Irregular aneurysm morphology | 58(47.9%) | 96(50.5%) | 0.656 | 37 |
| Admission maximum temperature (Celsius) | 37.268±1.274 | 37.164±1.036 | 0.430 | 33 |
| Acute hydrocephalus | 109(84.5%) | 200(91.3%) | 0.550 | 0 |
| ***Admission laboratory parameters**** | | | | |
| Leu(Leu/ul) | 14.345±5.292 | 14.868±5.130 | 0.377 | 18 |
| Hb(g/L) | 12.246±1.663 | 12.456±1.870 | 0.302 | 16 |
| CRP(mg/L) | 1.296±3.026 | 1.530±3.130 | 0.507 | 19 |
| cTnI( ng/L) | 170.741±945.690 | 33.588±153.326 | 0.250 | 167 |
| Mb(ug/L) | 230.579±258.412 | 703.718±4015.328 | 0.471 | 237 |
| LE(Leu/ul) | 14.026±5.266 | 14.697±5.336 | 0.320 | 77 |
| ESR(mm/h) | 4.254±0.427 | 4.285±0.568 | 0.612 | 77 |
| HCT(%) | 38.159±3.635 | 38.106±4.834 | 0.919 | 77 |
| MCV(fl) | 89.904±5.677 | 89.203±6.030 | 0.350 | 77 |
| MCH(pg) | 30.681±2.211 | 30.571±2.216 | 0.695 | 77 |
| MCHC(g/L) | 34.128±1.154 | 34.276±1.173 | 0.316 | 77 |
| Thyroxine(nmol/L) | 231.577±60.261 | 220.989±78.818 | 0.252 | 77 |
| TRH(uIU/mL) | 10.275±0.886 | 10.442±0.878 | 0.272 | 200 |
| Sodium(mmol/L) | 140.000±3.227 | 140.769±3.316 | 0.066 | 78 |
| Potassium(mmol/L) | 3.989±0.478 | 3.891±0.522 | 0.131 | 78 |
| Chloride(mmol/L) | 109.577±5.425 | 109.593±5.968 | 0.983 | 79 |
| Calcium(mmol/L) | 2.176±0.121 | 2.178±0.168 | 0.906 | 85 |
| Creatinine(mg/dL) | 0.899±0.146 | 0.990±0.428 | **0.012** | 78 |
| Glucose(mg/dL) | 152.73±50.146 | 176.10±51.887 | **0.001** | 166 |
| Phosphorus(mol/L) | 3.166±0.792 | 3.161±1.011 | 0.969 | 140 |
| GFR(mL/min) | 78.470±15.772 | 77.754±19.808 | 0.779 | 123 |
| ALB(g/dL) | 13.57±4.741 | 15.40±6.083 | **0.004** | 78 |
| CK(U/L) | 219.237±504.929 | 359.872±1580.752 | 0.395 | 79 |
| TBIl(μmol／L) | 0.564±0.289 | 0.573±0.306 | 0.803 | 78 |
| DBIL(μmol／L) | 0.311±0.369 | 0.305±0.336 | 0.924 | 226 |
| AST(U/L) | 39.062±34.637 | 41.690±44.935 | 0.618 | 77 |
| ALT(U/L) | 31.330±25.020 | 33.339±31.917 | 0.593 | 77 |
| GGT(U/L) | 43.309±88.533 | 52.414±131.951 | 0.544 | 77 |
| LDH(U/L) | 231.624±78.207 | 241.399±78.780 | 0.337 | 87 |
| ALP(U/L) | 71.620±29.429 | 74.884±44.453 | 0.579 | 148 |
| AMY(U/dl) | 27.690±21.737 | 25.178±12.297 | 0.331 | 112 |
| ACP(U/dl) | 6.342±0.584 | 6.229±0.858 | 0.202 | 78 |
| ***Adverse events during aSAH*** | | | | |
| Aneurysm rebleed | 4(3.1%) | 21(9.6%) | **0.024** | 0 |
| CNS Infection | 40(31.0%) | 63(29.3%) | 0.738 | 4 |
| Decompressive craniectomy | 42(32.6%) | 116(53.0%) | **<0.001** | 0 |
| Increased ICP | 63(48.8%) | 157(71.7%) | **<0.001** | 0 |
| TCD > 120m/s | 80(65.6%) | 105(56.5%) | 0.110 | 40 |
| Angiographic vasospasm | 28(21.7%) | 60(27.4%) | 0.238 | 0 |
| Days with fever | 6.248±3.806 | 6.441±4.055 | 0.673 | 25 |
| Systemic infection | 70(57.4%) | 121(61.1%) | 0.508 | 28 |
| Sepsis | 4(3.3%) | 16(8.1%) | 0.148 | 28 |
| Pneumonia | 38(31.1%) | 83(41.9%) | 0.054 | 28 |
| Pleural effusion | 1(0.8%) | 6(2.8%) | 0.215 | 4 |
| Pneumothorax | 4(3.2%) | 7(3.2%) | 0.985 | 4 |
| Tracheotomy | 4(3.2%) | 5(2.3%) | 0.622 | 4 |
| Bacteremia | 4(3.3%) | 13(6.6%) | 0.203 | 28 |
| Acute coronary syndrome | 3(2.6%) | 10(5.5%) | 0.229 | 48 |
| New onset of arrhythmia | 6(5.1%) | 17(9.2%) | 0.195 | 46 |
| Thromboembolic complications | 5(4.0%) | 5(2.3%) | 0.373 | 4 |
| Gastro-intestinal complication | 0(0.0%) | 3(1.4%) | 0.186 | 4 |
| Liver dysfunction | 8(6.8%) | 23(12.0%) | 0.138 | 38 |
| Epilepsy | 40(31.0%) | 53(24.2%) | 0.166 | 0 |
| Seizure at onset | 16(12.4%) | 23(10.5%) | 0.587 | 0 |
| DCI infarction | 21(16.3%) | 89(40.8%) | **<0.001** | 1 |
| Early infarction | 30(23.3%) | 122(56.0%) | **<0.001** | 1 |
| ** Leu: leucocyte, Hb: Hemoglobin, CRP: C-reactive protein, cTnI: cardiac troponin, Mb: Myoglobin, LE: leukocyte esterase, ESR: erythrocyte sedimentation rate, HCT: hematocrit, MCV: mean corpuscular volume, MCH: mean corpuscular hemoglobin, MCHC: mean corpuscular hemoglobin concentration, TRH: thyrotropin-releasing hormone, GFR: glomerular filtration rate, ALB: albumin, CK: creatine kinase, TBIl: total bilirubin, DBIL: direct bilirubin, AST: aspartate aminotransferase, ALT: alanine aminotransferase, GGT: γ-glutamyl transpeptidase, LDH: Lactic De- Hydrogenase, ALP: alkaline phosphatase, AMY: amylase, ACP: acid phosphatase.* | | | | |

**Supplementary Table 3** The cumulative impact of dilated pupil(s), cardiac valve disease, and age > 55 years on outcome

| No. of risk factors  (Pupil;Cardiac;Age) | No. of Patients in Subgroup | 6-month mRS  ≥2  N (%) | 6-month mRS  ≥3  N (%) | In Hospital mortality  N (%) |
| --- | --- | --- | --- | --- |
| 0 | 156 | 96 (61.5) | 78 (50.0) | 34 (21.8) |
| 1 | 144 | 109 (75.7) | 94 (68.8) | 39 (27.1) |
| 2 | 44 | 39 (88.6) | 38 (86.4) | 20 (45.5) |
| 3 | 4 | 4 (100) | 4 (100) | 2 (50.0) |

**Supplementary Fig. 1** ROC Curves and Youden Index for Creatinine, Glucose, and Albumin

**
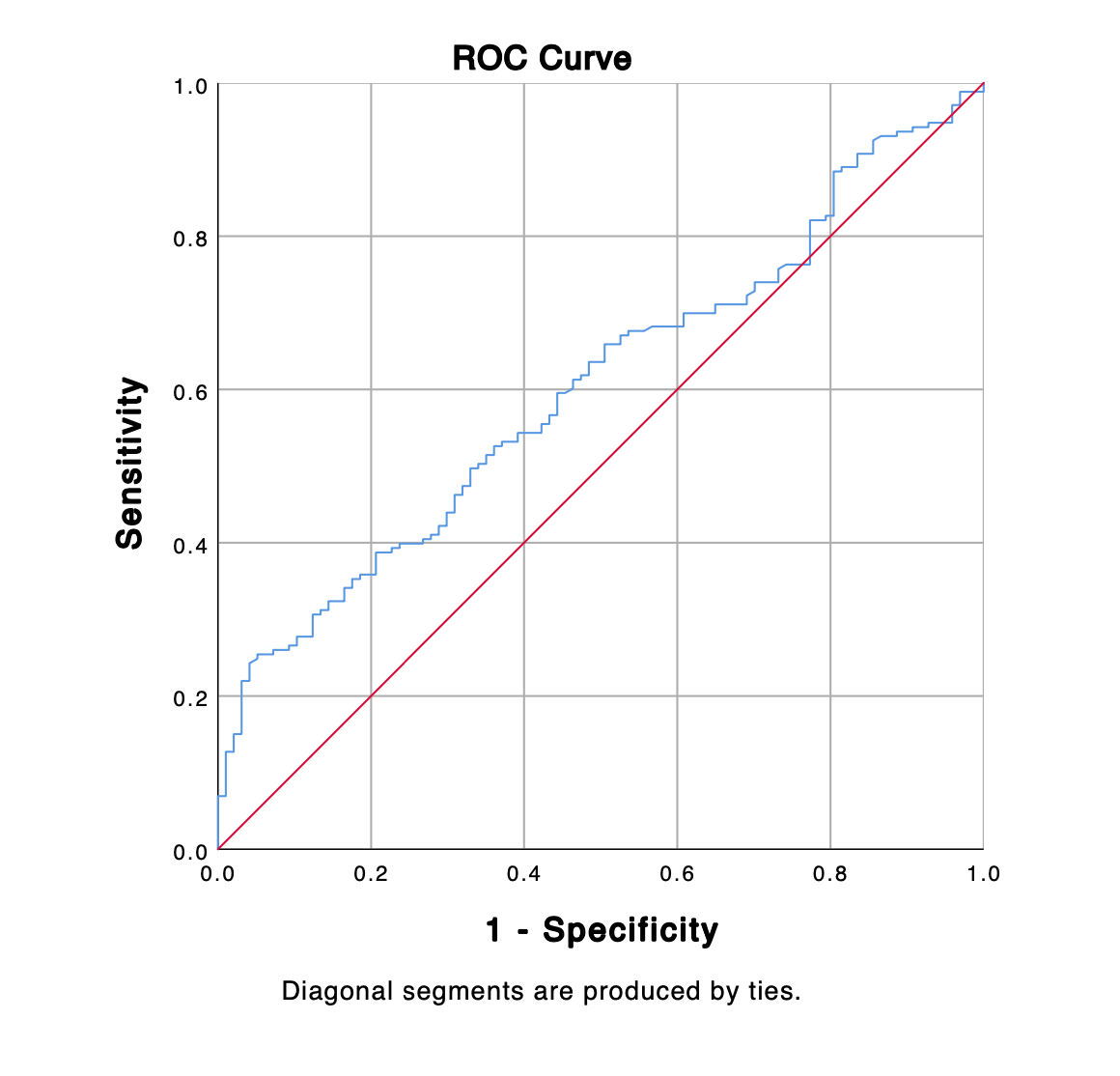
**

AUC=0.603

1.002 (0.052, 0.254)

a. ROC curve for Creatinine

**
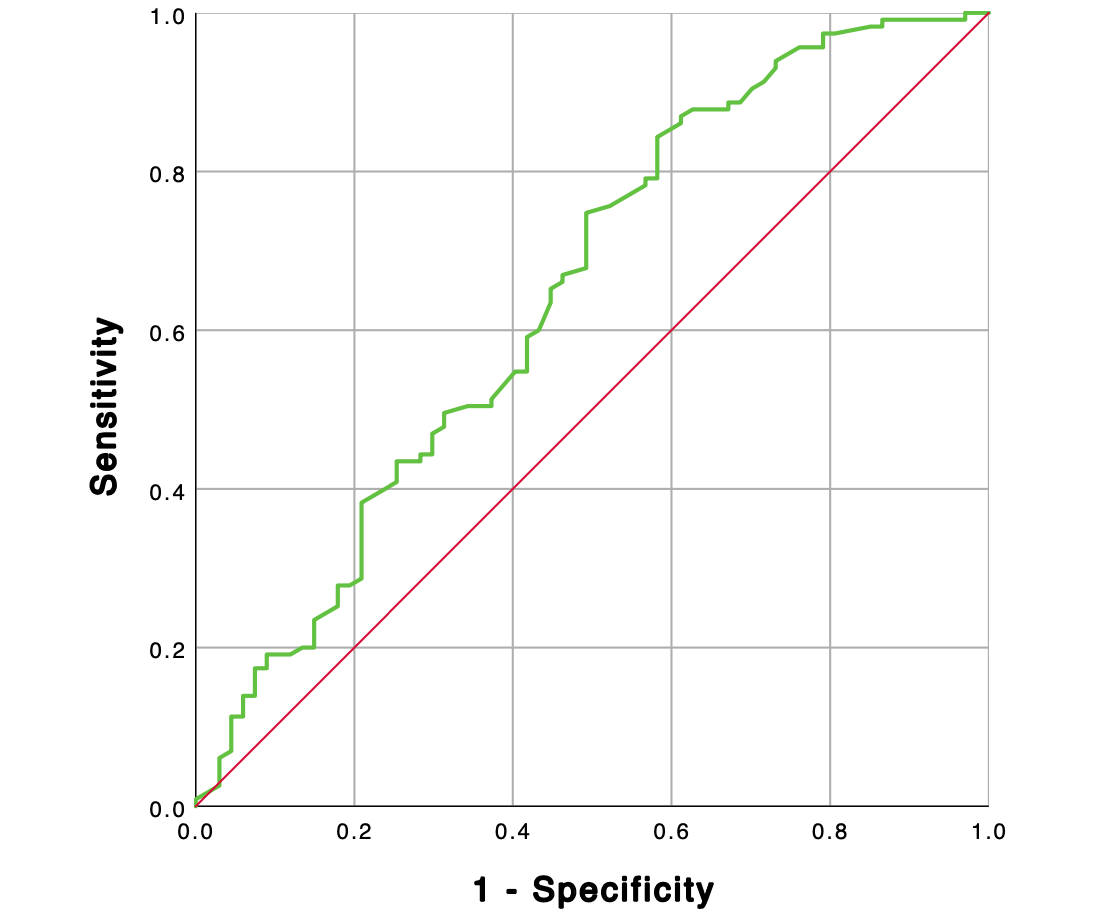
**

AUC=0.645

127.5 (0.582, 0.843)

b. ROC curve for Glucose

**
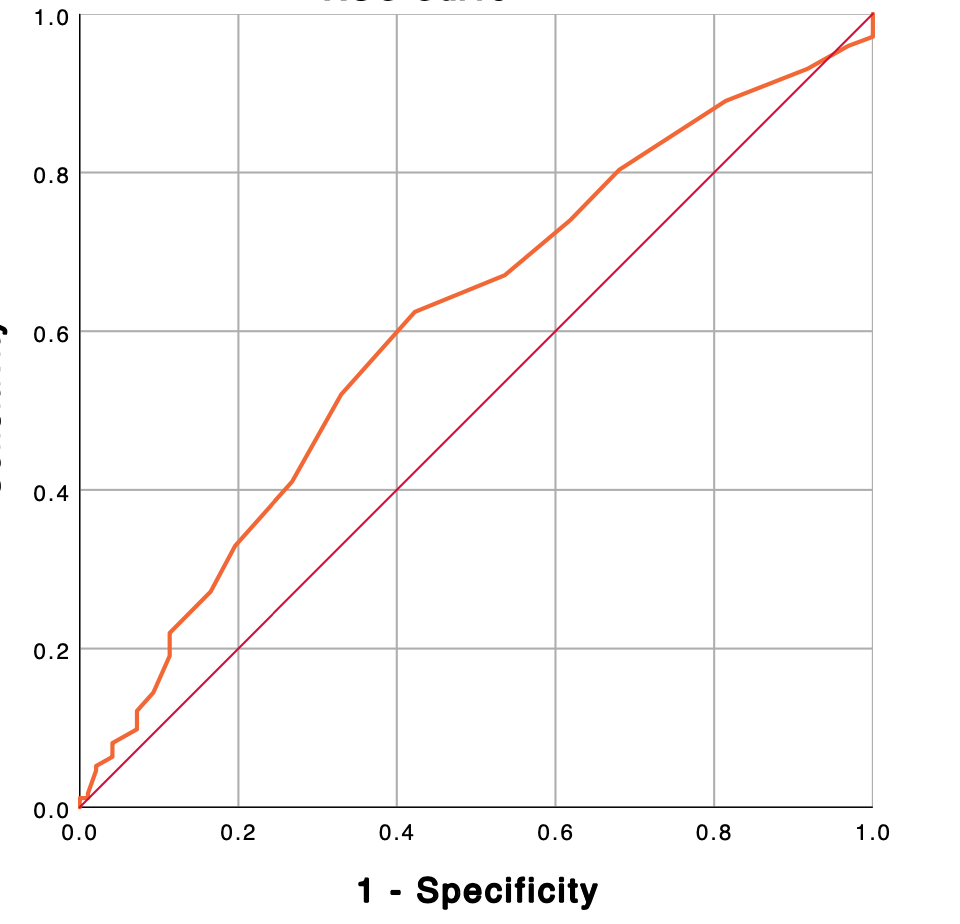
**

AUC=0.606

13.5 (0.423, 0.624)

c. ROC curve for Albumin

*The ROC curves illustrate the predictive value of creatinine, glucose, and albumin for unfavorable outcomes. The optimal thresholds were determined using the Youden index, as shown by the points. Corresponding AUC values are reported in the figure.*

**Supplementary Fig. 2** Impact of Risk Factors on Different Outcomes


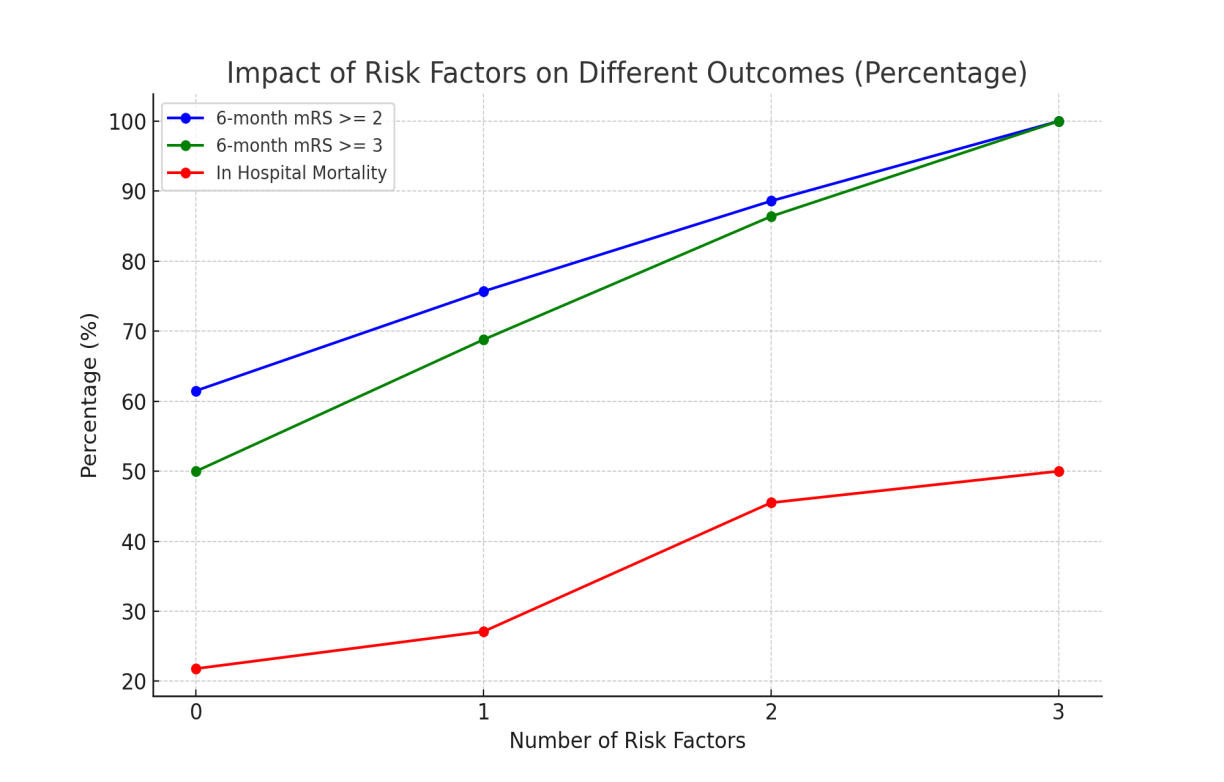

Supplement: Supplementary file 1 — (DOCX 440 KB) [file 10143_2025_3188_MOESM1_ESM.docx]
